# The Impact of Early Life Stress on Growth and Cardiovascular Risk: A Possible Example for Autonomic Imprinting?

**DOI:** 10.1371/journal.pone.0166447

**Published:** 2016-11-18

**Authors:** Reiner Buchhorn, Sebastian Meint, Christian Willaschek

**Affiliations:** Department of Pediatrics, Caritas Krankenhaus, Bad Mergentheim, Germany; Technion Israel Institute of Technology, ISRAEL

## Abstract

**Introduction:**

Early life stress is imprinting regulatory properties with life-long consequences. We investigated heart rate variability in a group of small children with height below the third percentile, who experienced an episode of early life stress due to heart failure or intra uterine growth retardation. These children appear to develop autonomic dysfunction in later life.

**Results:**

Compared to the healthy control group heart rate variability (HRV) is reduced on average in a group of 101 children with short stature. Low HRV correlates to groups of children born small for gestational age (SGA), children with cardiac growth failure and children with congenital syndromes, but not to those with constitutional growth delay (CGD), who had normal HRV. Reduced HRV indicated by lower RMSSD and High Frequency (HF)-Power is indicating reduced vagal activity as a sign of autonomic imbalance.

**Conclusion:**

It is not short stature itself, but rather the underlying diseases that are the cause for reduced HRV in children with height below the third percentile. These high risk children—allocated in the groups with an adverse autonomic imprinting in utero or infancy (SGA, congenital heart disease and congenital syndromes)—have the highest risk for ‘stress diseases’ such as cardiovascular disease in later life. The incidence of attention deficit disorder is remarkably high in our group of short children.

## Introduction

Growth measurement is an easy accessible parameter and considered to be the best global single indicator of children's health. Impaired growth with strong evidence is associated with delayed mental development resulting in poorer school performance and lower intellectual achievement [1]. Additionally there is a widely described inverse association between height and ischemic heart disease. Genetic conditions seem to be of minor importance for individual growth. Genome-wide polymorphism analysis using data from 253,288 individuals has identified 697 genetic variants that together accounted for one-fifth of the heritability for adult height [2]. Epigenetic heredity thus appears to be A further important determinant of adult height may be found in epigenetic mechanisms[3]. A candidate to mediate information about environmental influence into epigenetic traits may be found in the modulation of DNA methylation. Human height is highly defined phenotype. Growth failure therefore seems to be a model for imprinting, probably due to epigenetic mechanisms [4]. Recently we published our model about early life stress to explain growth failure in children with growth hormone deficiency (GHD) and small for gestational age (SGA). In these children growth failure is related to sub sensitivity of the α2-adrenoreceptor. This impairment was measured by heart rate variability (HRV) analysis during clonidine testing. [5]. Our pathophysiological approach to evaluate the autonomic nervous system is deducted from HRV- analysis, a simple and effective tool to investigate heart-brain interaction. HRV analysis can be used for cardiovascular risk stratification [6]. Furthermore, there is an interaction between neurologic development and HRV: As we have shown recently attention deficit hyperactivity disorder (ADHD) is associated with reduced vagal tone measured by HRV analysis [7].

The environmental conditions within the first 1000 days after implantation in utero up to the second year of life have lifelong consequences for health [8]. Most of all nutrition and early life stress influence the risk of metabolic disease, such as diabetes mellitus and cardiovascular disease.

Intra uterine growth retardation and heart failure due to congenital heart disease are considered to be among the most stressful conditions during early life and are associated with growth restriction in later life. In order to investigate the effect of early life stress we measured autonomic function in later life in a group of small children with height below the third percentile.

## Methods

### Patients

For our analyses we subdivided a group of 101 consecutive children with height below the third percentile into the following diagnostic groups. All of them had been referred between the years 2005 to 2015 to the pediatric outpatient clinic of our hospital:

1. Congenital syndromes (N = 17) like Down's -, Turner-, Noonan-, Rett- and Silver Russell syndrome. These syndromes are known as genetic disorders with impact on height (genomic imprinting).
2. Small for gestational age (SGA; N = 30): SGA is due to malnutrition in utero (gestational imprinting). SGA is related to an enhanced risk of cardiovascular disease and ADHD.
3. Growth hormone deficiency (GHD; N = 11): GHD is related to an autonomic disorder in children and adults with a known enhanced cardiovascular risk.
4. Congenital heart disease (CHD; N = 10): CHD patients have an enhanced risk of growth failure and ADHD in later life related to heart failure in infancy (infant imprinting) [9, 10].
5. Constitutional growth delay (CGD; N = 25): CGD is characterized by delayed bone age maturation. These children are our additional "healthy control" to analyze the sole effect of short stature on the autonomic nervous system.
6. Miscellaneous (N = 8). These children cannot be assigned to the other groups. Most of these patients suffered from gastrointestinal disease like celiac disease.

Furthermore, we investigated the impact of early life stress on HRV in 20 infants with heart failure and 10 infants with failure to thrive in and ex utero in order to demonstrate the autonomic effects of these diagnoses during early life.

### Control group

Healthy children (N = 55) were collected from our “normal values heart rate variability project”, therefore data of patients, who attended our outpatient clinic for exclusion of cardiac arrhythmia were analysed retrospectively. The retrospective analysis was approved by the ethical board of our state’s medical chamber (Landesärztekammer Baden Württemberg) and recently published [11]. Children with a body mass index above 96% and height below 3% were excluded from this group of healthy children.

### 24 hour ECG and analysis of heart rate variability

Measurement and interpretation of HRV parameters in the current sample were standardized according to the Task Force Guidelines [12]. Cardiac autonomic functioning was measured by a 24 hour Holter 12 bit digital ECG (Reynolds Pathfinder II, Spacelabs, Germany; 1024 scans/sec). Daytime and night time periods were defined according to patient’s protocols. All Holter recordings were reviewed by the same experienced cardiologists (RB and CW) and were edited to validate the systems QRS labelling. Measures of HRV were calculated employing only normal-to-normal intervals. QRS-complexes classified as noise were ejected. A minimum of 23 hours of analyzable data and minimally 95% of analyzable NN intervals were required for the data to be included. For time domain measures, mean RR interval, resulting heart rate and the following HRV parameters were calculated as average hourly values and as 24-hour average values: square root of the mean of the sum of squares of differences between adjacent NN-intervals (rMSSD) and the standard deviation of NN intervals (SDNN). RMSSD predominantly reflects changes in vagal tone. SDNN—this parameter reflects global heart rate variability—is dually influenced by cholinergic and adrenergic activity, as well as other physiological inputs. In the following we use the term vagal tone for the part of the autonomic nervous system measured by rMSSD. For frequency domain measures, beat-to-beat fluctuations were transformed to the frequency domain using Fast Fourier Transformation. Spectral power was determined over three frequency regions of interest: Very low frequency (VLF, < 0.04 Hz), low frequency (LF, 0.04–0.15 Hz) and high frequency (HF, 0.15–0.4 Hz) with derived HF/LF ratio. LF reflects mostly sympathetic activity, HF vagal tone. The methods of 24 hour ECG and analysis of heart rate variability have been described earlier [5].

### Statistical Analysis

Groups were compared with controls by means, standard deviations and t-testing.

P-values below 0.005 were considered as statistically significant.

From anthropometrical data percentile values and BMI have been calculated using the free myBMI web page (nowadays changed to myBMI4Kids) according to percentile data by Kromeyer-Hauschild et al.

### Ethics

The FDA issued a Safety Alert in August 2011, reporting that a French study concluded that people with certain types of short stature treated with recombinant human growth hormone during childhood and who were then followed over a long period of time, were at a small increased risk of death when compared to individuals in the general population of France. This increased mortality rate is related to cardiovascular events [13] and/or stroke [14]. We routinely communicate this enhanced cardiovascular risk to all parents prior to growth hormone treatment and propose a monitoring of cardiovascular risk by 24 hour Holter ECG and NT-BNP measurements as part of routine blood tests for short stature. We received an informed consent from nearly all parents of children with growth failure, but we certainly disregard performing these examinations if the parents refuse. All parents and patients (if appropriate according to patient`s age) enrolled in this analysis have given oral informed consent.

## Results

Routinely measured clinical and laboratory data are illustrated in Table 1. Compared to an age matched healthy control group mean height is significantly reduced with an SDS about—2.5, with very low values in children with congenital syndromes. Bodyweight and body mass index are normal on average in all groups. As expected birth weight is low in children with SGA and congenital syndromes. Insulin like growth factor 1 (IgF1) and insulin like growth factor binding protein (IgFPB3) are in the low normal range in children with growth failure from different aetiologies. NT-BNP is elevated in children with congenital heart disease but also in more than 50% of children with SGA and GHD. Patients in the miscellaneous group were significantly older than controls (p < 0.0001). Because HRV is an age related parameter further statistical analysis of HRV data from this group was not performed.

**Table 1:** Anthropometric data and laboratory of study groups.

Parameter	Healthy Control	All GF	CGD	SGA	Cardiac GF	Syndromes	GHD	Miscellaneous
**N**	55	101	25	30	10	17	11	8
**Age [years]**	7.5 ± 2.1	8.0 ± 4.3	7.8 ± 2.8	7.4 ± 4.1	9.6 ± 5.1*	7.8 ± 6.0	7.1 ± 3.1	11.2 ± 5.0***
**Height [cm]**	125 ± 14	114 ± 23***	114 ± 16***	111 ± 22***	121 ± 24	108 ± 31***	110 ± 18**	134 ± 30
**Height SDS**	-0.1 ± 0.9	-2.6 ± 0.9	-2.5 ± 0.9	-2.4 ± 0.8	-2.5 ± 1.0	-3.2 ± 1.2	-2.5 ± 0.6	-2.2 ± 0.4
**Weight [kg]**	38.8 ± 21.4	38.1 ± 3.2	38.3 ± 2.3	36.6 ± 3.7	40.7 ± 0.8	37.7 ± 2.4	40.2 ± 3.3	38.8 ± 1.1
**BMI [%]**	38.3 ± 22.5	34.2 ± 28.8	37.8 ± 25.6	33.6 ± 30.7	17.7 ± 21.8**	34.5 ± 40.6	32.5 ± 21.6	47.9 ± 33.2
Data without statistical analysis because of missing data in the healthy control group **(Given data from literature)**								
**Birthweight [g]**	> 2500	2696 ± 855	3032 ± 776	2014 ± 699	3207 ± 454	2514 ± 639	3434 ± 594	3342 ± 208
**GA [weeks]**	37–41	38.1 ± 3.1	38.3 ± 2.3	36.6 ± 3.7	40.6 ± 0.8	37.7 ± 2.4	40.1 ± 3.3	39.8 ± 1.1
**IgF1 [ng/ml]**	110–600	89 ± 59	80 ± 39	96 ± 74	67 ± 43	74 ± 13	103 ± 74	
**IgFPB3 [ng/ml]**	1300–4700	3029 ± 1093	3028 ± 1103	3135 ± 887	2720 ± 1570	3079 ± 1658	2996 ± 1222	
**NT- BNP [pg/ml]**	52 (10–157)	182 ± 197	86 ± 60	166 ± 156	405 ± 338	108 ± 70	159 ± 137	

GF = Growth Failure; CGD = Constitutional Growth Delay; SGA = Small for Gestational Age; GF = Growth Failure; GHD = Growth Hormone Deficiency; GA: gestational age; IgF1: Insulin like growth factor 1; IgFPB3: Insulin like growth factor 1 binding protein; NT-Pro-BNP: brain natriuretic peptide. T-test between control and patient groups:

*P-value < 0.005;

** P-value < 0.001;

***P-value < 0.0001

Compared to the control group HRV measured by rMSSD in the 101 children with height below the third percentile is reduced on average by 35.3± 12.2 msec versus 42.5 ± 10.9 p < .0001 (Table 2). However, low HRV corresponds to the groups with cardiac growth failure, congenital syndrome and those which are small for gestational age, but not to children with constitutional growth delay who have normal HRV. Children with growth hormone deficiency have isolated reduced SDNN values. Mean heart rates are elevated in children with SGA and congenital syndromes but not in children with cardiac growth failure. Autonomic imbalance in children with short stature largely relates to low vagus activity indicated by significantly lower RMSSD and HF-Power in children with SGA, cardiac growth failure and congenital syndromes. Vagus activity is normal in children with constitutional growth delay.

**Table 2:** 24 hour HRV analysis of study groups and controls.

Parameter	Healthy Control	All GF	CGD	SGA	Cardiac GF	Syndromes	GHD
N	55	101	25	30	10	17	11
**24—hour HRV**							
**HR [bpm]**	90.1 ± 8.4	**95.7 ± 14.2**^b^	92.6 ± 9.1	**97.3 ± 10.8**^c^	90.5 ± 19.9	**104.9 ± 20.1**^c^	92.6 ± 9.1
**SDNN [ms]**	142 ± 36	**123 ± 43**^b^	136 ± 23	**124 ± 28**^a^	125 ± 90	**98 ± 42**^d^	**110 ± 20**^b^
**RMSSD ms]**	42.5 ± 10.9	**35.3 ± 12.2**^c^	39.5 ± 10.4	**35.7 ± 10.1**^b^	**24.6 ± 12.9**^c^	**30.8 ± 14.9**^b^	39.5 ± 10.4
**TP [ms**^**2**^**]**	4857 ± 2237	**3858 ± 2553**^a^	4224 ± 1869	4256 ± 2370	3464 ± 4452	**2881 ± 2687**^b^	3523 ± 1442
**VLF [ms**^**2**^**]**	2692 ± 1589	**2190 ± 1849**	2284 ± 1232	2458 ± 1782	2414 ± 3627	**1554 ± 1539**^a^	1759 ± 760
**LF [ms**^**2**^**]**	1284 ± 581	**1002 ± 612**^b^	1122 ± 512	1097 ± 555	**752 ± 783**^a^	**825 ± 792**^a^	987 ± 553
**HF [ms**^**2**^**]**	775 ± 301	**577 ± 335**^c^	719 ± 326	**599 ± 326**^a^	**249 ± 155**^c^	**416 ± 388**^c^	686 ± 238
**HF/LF**	0.65 ± 0.22	0.63 ± 0.28	0.69 ± 0.28	0.58 ± 0.24	0.5 ± 0.31	**0.47 ± 0.17**^b^	0.81 ± 0.35
**Nighttime—HRV**							
**HR [bpm]**	76.5 ± 8.5	**82.3 ± 15.4**^a^	78.3 ± 7.0	**81.4 ± 11**^a^	80.9 ± 22.9	**96.2 ± 24.5**^c^	81.3 ± 6.9
**SDNN [ms]**	81 ± 26	77 ± 52	100 ±83	78 ± 32	**55 ± 48**^a^	**56 ± 34**^b^	70 ± 13
**RMSSD [ms]**	65.7 ± 25.4	**56.2 ± 28.0**^a^	66.1 ± 23.3	61.4 ± 29.5	**33.6 ± 32.8**^c^	**41.8 ± 28.6**^b^	63.2 ± 21.1
**TP [ms**^**2**^**]**	6480 ± 3426	5363 ± 4130	6051 ± 2982	5768 ± 4095	4901 ± 7885	**3896 ± 4055**^a^	4617 ± 1504
**VLF [ms**^**2**^**]**	3564 ± 2539	3035 ± 3257	3244 ± 2244	3238 ± 3274	3609 ± 6855	2138 ± 2326	2311 ± 1050
**LF [ms**^**2**^**]**	1642 ± 876	**1326 ± 889**^a^	1557 ± 767	1431 ± 897	**914 ± 1057**^a^	1078 ± 1212	1235 ± 523
**HF [ms**^**2**^**]**	1185 ± 473	**911 ± 552**^a^	1162± 500	978 ± 598	**336 ± 282**^c^	**609 ± 600**^c^	981 ± 343
**HF/LF**	0.86 ± 0.45	0.79 ± 0.48	0.84 ± 0.37	0.82 ± 0.52	0.56 ± 0.37	0.57 ± 0.34^a^	0.97 ± 0.7

8 patients (the miscellaneous group) are not shown in the table.

GF = Growth Failure; CGD = Constitutional Growth Delay; SGA = Small for Gestational Age; GF = Growth Failure; GHD = Growth Hormone Deficiency

SDNN: Standard deviation of all NN intervals; RMSSD: The square root of the mean of the sum of the squares of differences between adjacent NN intervals; TP: Total Power; VLF: Very low frequency power; LF: Low frequency power; HF: High frequency power; HF/LF: Ratio HF to LF. T-test between healthy control and patient groups:

^a^ P-value < 0.005;

^b^ P-value < 0.001;

^c^ P-value < 0.0001

^**d**^ P-value < 0.00001

There was a remarkably high number of patients with the additional diagnosis of ADHD (24; 23.8%) in our study group. Statistical subgroup analysis showed no significant difference between short children according to the diagnosis of ADHD or concomitant treatment with methylphenidate.

**Fig 1:**
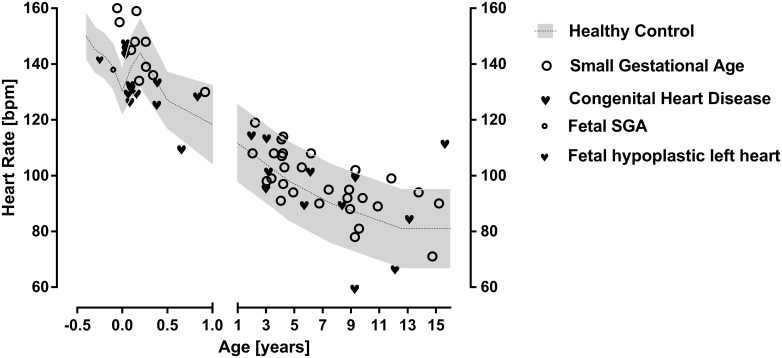
24 hour mean heart rate. 24 hour mean heart rate in small children with heart failure due to congenital heart disease or small for gestational age compared to controls. Additionally (left side) we provide data of a group of congenital heart disease (N = 20) and SGA infants (N = 10) and fetal data (from literature).

**Fig 2:**
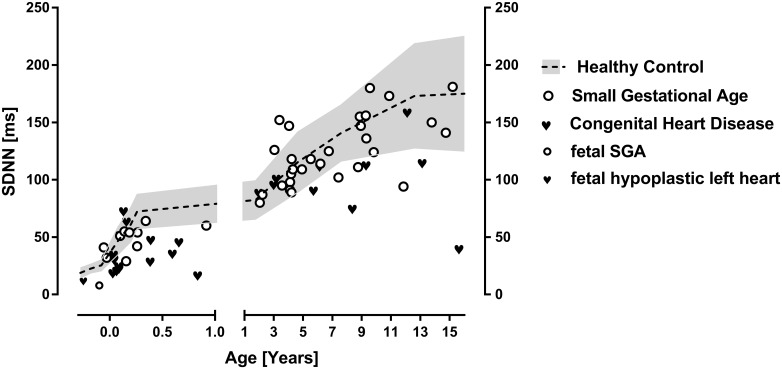
Global heart rate variability. Indicated by the HRV parameter SDNN in children with heart failure due to congenital heart disease or small for gestational age compared to controls. Additionally (left side) we provide data of a group of infants with congenital heart disease (N = 20) and SGA infants (N = 10) and fetal data (from literature).

**Fig 3:**
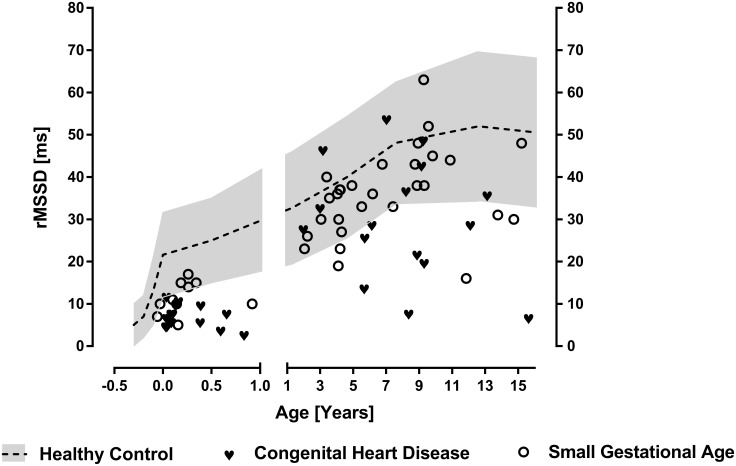
Vagus activity indicated by the HRV parameter rMSSD. RMSSD in infants with heart failure due to congenital heart disease or small for gestational age compared to controls. Additionally (left side) we provide data of a group of infants with congenital heart disease (N = 20) and SGA infants (N = 10)

We further illustrate data of infants with heart failure due to congenital heart disease (N = 20) indicated by very high NT-Pro-BNP value of 16437 ± 7066 pg/l. Additionally we illustrate data of infants born small for gestational age and with prematurity (N = 10) with a birth weight of 1176 ± 552 g and 31 ± 5 weeks gestational age on average. These data clearly illustrate normal heart rates (Fig 1) despite severe heart failure and prematurity. However these groups of infants show highly reduced global HRV indicated by SDNN (Fig 2) and rMSSD (Fig 3) in infancy. Interestingly the mean NT-BNP of 822 ± 360 pg /l in small for gestational age infants is elevated too.

## Discussion

### Autonomic imbalance in children with symptomatic short stature

Autonomic imbalance measured by HRV is a predictor of metabolic risk, cardiovascular disease, diabetes and early mortality [5]. We now clearly demonstrate a significant autonomic imbalance in children with short stature due to a history of small for gestational age, congenital syndromes and congenital heart disease. These children have a well-known enhanced cardiovascular risk.

Remarkably different patient groups show different patterns of autonomic imbalance. Increased heart rate is found in the SGA and the congenital syndromes group. Reduced SDNN is found in the syndromes and in the growth hormone deficiency group. RMSSD reduction can be diagnosed in the SGA, the cardiac growth failure and the syndromes group. HF component is reduced in the cardiac growth failure and the syndromes group. These different patterns of autonomic imbalance suggest the affection of different regulatory properties or different types of lesions.

Because HRV measurement can be performed and repeated easily this method provides the possibility of monitoring individual cardiovascular risk at an early stage as well as the impact of pharmacological treatment such as growth hormone. It may be used as an additional diagnostic tool combined with measurement of blood pressure, body weight or serum lipids in order to asses risk stratification. Moreover our data clearly indicate that short stature per se is not a factor for an autonomic imbalance or stress disease as shown in 25 children with constitutional growth delay. We agree with DE Sandberg et al. [15] that short stature as an isolated physical characteristic is no cause of psychological diseases. The impression of an association between short stature and psychological problems has been based largely upon methodologically weak studies that have typically confounded short stature with other medical and neurocognitive problems due to the underlying diseases like small for gestational age, congenital syndromes, congenital heart disease and probably prematurity.

Focusing on the group of SGA patients we provide evidence for autonomic imbalance in our group of patients, which is described by ongoing growth failure—more precisely by the lack of catch up growth. An adult group of SGA in the study of Schäffer et al., which showed normal growth, was autonomically healthy [16]. So the individual ability of an SGA patient to generate catch up growth seems to be related to the integrity of autonomic regulation. By this the lack of catch up growth seems to be a clinical marker for cardiovascular risk within this group of patients.

### Early life stress in children with SGA or heart failure

With respect to our groups of patients with heart failure and SGA we are investigating patients who experienced severe stress during different episodes in early life. SGA patients are most likely to have experienced caloric restriction in utero resulting in intrauterine growth restriction. The group of SGA premature infants shows SDNN and rMSSD in the lowest range (Figs 2 and 3). From animal models there are data from literature describing the impact of intrauterine nutrition on an individual’s regulatory properties mainly on the level of hypothalamic regulation [17, 18]. In accordance with Barker’s hypothesis our data indicate that birth weight is an important predictor for autonomic imbalance in later life.

Referring to the heart failure patients there has to be recognized an episode of severe stress resembled by well-known activation of neuro humeral axis, release of renin, cortisol and norepinephrine. Heart failure in this group of infants was severe indicated by extraordinary NT-Pro-BNP value of 16437 ± 7066 pg/l. As shown in Figs 2 and 3 infants with early life stress due to heart failure or SGA have reduced rMSSD in infancy and most of these children with height below the third percentile in later life further have low rMSSD values. Recently we published longitudinal data that show that reduced HRV in later life due to congenital heart disease may be related to reduced HRV in early infancy [19].

As shown in the literature there seems to be a common pathway resulting from early life stress leading to elevation of NT-Pro-BNP. This constellation is shown in children with failure to thrive [20] as well as in infants with heart failure [21] but also in infants with small gestational age at birth [22]. Circulating norepinephrine levels in girls with SGA are elevated and the renin angiotensin aldosterone system in boys with SGA is activated. These hormonal changes correlate with blood pressure at the age of 10 years [23].

### A possible example for autonomic imprinting?

We speculate to encounter here an underlying mechanism of early life stress which is imprinting the development of impaired autonomic regulation. This idea may explain concordant effects of nutrition and stress on metabolic regulation, growth and neurodevelopment:

1. We have shown recently that nutrition has prominent and reproducible effects on the autonomic nervous system [24].
2. Heart failure in infancy like other severe diseases causes sympathetic activation, suppresses parasympathetic tone and is related to growth failure, as well as attention deficit disorder [19].

A. Waterland and C. Garza defined the term metabolic imprinting as a “basic biological phenomenon that putatively underlies relations between nutritional experiences during early life and later diseases” [25]. For their definition the following conditions are characterized:

1. “a susceptibility limited to a critical ontogenetic window early in development” (the critical period)
2. “a persistent effect lasting through adulthood” (autonomic disorder)
3. “a specific and measurable outcome” (height, cognition, cardiovascular disease), and
4. “a dose-response or threshold relation between a specific exposure and outcome”.

There is a clear historical precedent for this definition: Konrad Lorenz chose “imprinting” to refer to the setting of certain animalistic behavior that resulted from early experience. Central to his definition was the fact that imprinting may only occur during “a narrowly defined period in the individual’s life” (the critical period), moreover that the imprinted behavior cannot be ‘forgotten’.

We assume different critical periods in early life for autonomic imprinting:

1. **Genomic imprinting** seems to be an epigenetic phenomenon most of all in some congenital syndromes. One well known mechanism is the parent-of-origin-specific genomic imprinting. If the allele inherited from the father is imprinted only the allele from the mother is expressed and vice versa.
2. **Gestational imprinting** mainly by fetal malnutrition results in a baby small for gestational age. Barker was the first who used the term fetal programming in his hypothesis [26].
3. **Neonatal imprinting** during the first 4 weeks of life occurs mostly due to severe disease like premature birth or feeding problems.
4. **Infant imprinting** during the first year of life is greatly due to severe disease like heart failure. Preoperative heart failure is one of the most stressful life events in infancy and has been neglected as a well-defined early life stress model with longtime consequence like growth failure and ADHD. We use this model owing to the fact that in most infants stress is limited to a well-defined time period and is terminated by an operative procedure at a distinct time point (predominantly with an age of approximately 6 month in heart defects with left-to-right shunts) [24].
5. **Childhood imprinting** during early childhood due to severe disease, neglect, trauma and abuse.
6. We are fortunate to have only a small number of children with short stature owing to starvation in Germany. However in 2010, 171 million children under 5 years of age suffer from stunting globally, with 98% being from low- and middle-income countries. Weight gain in the first two years of life is an important predictor of schooling outcomes in pooled analyses from five birth cohorts in these countries [1]. The understanding of child growth patterns is critical to the development and evaluation of appropriate interventions. Providing small-quantity lipid based nutrient supplements significantly increased growth and reduced stunting, wasting and anemia prevalence in young burkinabe children [27].

By understanding autonomic imprinting we are aware of the detrimental longtime effects of early life stress on growth and cardiovascular risk. Remarkable in our data is the high incidence of ADHD suggesting a relationship between symptomatic short stature and cognitive and behavioral properties. Improving care and nutrition for mothers and children within the first 1000 days after conception is one of the most promising public health policies [8].

Moreover if early life stress is not preventable due to severe diseases like heart failure or SGA, we may be able to improve long term outcome by targeted treatment of stress with beta blockers or supplementation with omega-3-fatty acids. Measurement of HRV as outlined here may help to monitor and evaluate these treatment options during further trials.

However, our data from a small pediatric department are observational data from a small group of patients. Further prospective clinical studies will be needed to identify critical periods of infant development and to evaluated treatment strategies.
